# Anatomical Location of the Vesical Branches of the Inferior Hypogastric Plexus in Human Cadavers

**DOI:** 10.3390/diagnostics14080794

**Published:** 2024-04-10

**Authors:** Emily P. Day, Benjamin R. Johnston, Stanley F. Bazarek, Justin M. Brown, Nucelio Lemos, Eve I. Gibson, Helaina N. Hurban, Susan B. Fecho, Lewis Holt-Bright, Daniel D. Eun, Michel A. Pontari, Elise J. De, Francis J. McGovern, Michael R. Ruggieri, Mary F. Barbe

**Affiliations:** 1 MD Program, Drexel University College of Medicine, Philadelphia, PA 19129, USA; epd45@drexel.edu; 2 Center for Translational Medicine, Lewis Katz School of Medicine of Temple University, Philadelphia, PA 19140, USA; evieg278@gmail.com (E.I.G.); or mruggieri@mgh.harvard.edu (M.R.R.); 3 Department of Neurosurgery, Brigham and Women’s Hospital, Boston, MA 02115, USA; bjohnston2@mgb.org (B.R.J.); sbazarek@bwh.harvard.edu (S.F.B.); 4 Neurosurgery Paralysis Center, Department of Neurosurgery, Massachusetts General Hospital, Boston, MA 02115, USA; jmbrown@mgh.harvard.edu; 5 Department of Obstetrics and Gynecology, University of Toronto Temerty Faculty of Medicine, Toronto, ON M5S 1A8, Canada; nucelio.lemos@utoronto.ca; 6 MD Program, Lewis Katz School of Medicine of Temple University, Philadelphia, PA 19140, USA; helaina.hurban@temple.edu; 7 School of Visual, Performing and Communication Arts, Barton College, Wilson, NC 27893, USA; susanfecho@gmail.com; 8 Aging + Cardiovascular Discovery Center, Lewis Katz School of Medicine of Temple University, Philadelphia, PA 19140, USA; holtbright@temple.edu; 9 Robotic Surgical Services, Lewis Katz School of Medicine of Temple University, Philadelphia, PA 19140, USA; daniel.eun@tuhs.temple.edu; 10 Department of Urology, Lewis Katz School of Medicine of Temple University, Philadelphia, PA 19140, USA; michel.pontari@tuhs.temple.edu; 11 Department of Urology, Albany Medical Center, Albany, NY 12208, USA; dee@amc.edu; 12 Department of Urology, Massachusetts General Hospital, Boston, MA 02115, USA; fmcgovern@mgh.harvard.edu; 13 Department of Biomedical Education and Data Science, Lewis Katz School of Medicine of Temple University, Philadelphia, PA 19140, USA

**Keywords:** bladder, pelvic nerve, pelvic ganglion, ureter

## Abstract

We have demonstrated in canines that somatic nerve transfer to vesical branches of the inferior hypogastric plexus (IHP) can be used for bladder reinnervation after spinal root injury. Yet, the complex anatomy of the IHP hinders the clinical application of this repair strategy. Here, using human cadavers, we clarify the spatial relationships of the vesical branches of the IHP and nearby pelvic ganglia, with the ureteral orifice of the bladder. Forty-four pelvic regions were examined in 30 human cadavers. Gross post-mortem and intra-operative approaches (open anterior abdominal, manual laparoscopic, and robot-assisted) were used. Nerve branch distances and diameters were measured after thorough visual inspection and gentle dissection, so as to not distort tissue. The IHP had between 1 to 4 vesical branches (2.33 ± 0.72, mean ± SD) with average diameters of 0.51 ± 0.06 mm. Vesical branches from the IHP arose from a grossly visible pelvic ganglion in 93% of cases (confirmed histologically). The pelvic ganglion was typically located 7.11 ± 6.11 mm posterolateral to the ureteral orifice in 69% of specimens. With this in-depth characterization, vesical branches from the IHP can be safely located both posterolateral to the ureteral orifice and emanating from a more proximal ganglionic enlargement during surgical procedures.

## 1. Introduction

Spinal cord injuries (SCI) induce voiding dysfunction of the bladder that includes retention, urine stasis, infection, and incontinence, and generally lead to decreased quality of life for SCI patients [1,2]. Upper motor neuron lesions (e.g., with cervical SCI) generally result in spastic bladders as the sympathetic tone is interrupted and local reflex arcs in the conus trigger dyssynergy. In contrast, lower motor neuron lesions result in flaccid paralysis of the bladder and happen after sacral spinal root or cauda equina injury with trauma or sacral chordomas. With either type of injury (upper or lower motor neuron), changes in bladder function can drastically change a patient’s quality of life [1,2]. Thus, reinnervation strategies are crucial for either patient population.

As suggested above, the bladder receives significant motor innervation from the spinal cord. Sacral spinal cord regions (primarily S2-4) give rise to pre-ganglionic parasympathetic motor axons called pelvic splanchnic nerves [3]. These parasympathetic pelvic splanchnic nerves join inferior branches of the hypogastric nerves to form the inferior hypogastric plexus (IHP, also termed the pelvic plexus [4]), located on both the right and left sides of the pelvis [5,6,7,8]. Sympathetic sacral splanchnic nerves from sacral located sympathetic ganglia also send branches into the IHP [9,10,11]. The anterior division of the IHP supplies nerve fibers to the bladder and ureter (i.e., vesical branches of the IHP, also known as vesical branches of the pelvic nerve [12,13,14,15,16], anterior branches of the IHP [9,17,18], and bladder nerve branches [10]). The anterior division of the IHP also supplies nerve branches to the uterus and vagina, or prostate [7]; see [9,10,11] for more regarding the anatomy of this entire plexus. The pre-ganglionic parasympathetic motor axons to the bladder synapse on post-ganglionic parasympathetic motor neurons in pelvic ganglia located near yet external to the bladder wall. These neurons then send axons via the vesical nerves to the intramural ganglia located in the bladder wall itself, which then innervate detrusor smooth muscle fibers [19,20].

Our pre-clinical canine studies over the past 18 years have sought to develop surgical approaches to reinnervate the bladder following a lower motor neuron lesion that led to a flaccid bladder. In the canine model, a lower motor neuron lesion with a flaccid bladder was induced by transecting the sacral ventral roots that induce bladder contractions upon intra-operative electrical stimulation. Functional reinnervation was restored by transferring a somatic peripheral nerve (donor choices have included obturator, femoral, sciatic, and genitofemoral nerve branches) to vesical nerves located adjacent to or on the bladder wall, which is the distal end of the anterior division of the inferior hypogastric plexus (IHP) [21,22,23]. The rationale for a somatic nerve transfer approach is to bypass sacral injuries by re-routing nerves originating from more cranially located un-injured spinal cord regions (e.g., lumbar) to the now de-sacralized vesical nerves. In our canine studies, we have documented successful functional reinnervation of the bladder using somatic nerve transfer to the vesical branches of the IHP [21,22,23] (renamed from “vesical branches of the pelvic nerve” to better match the current naming of these nerves and plexus in the clinical literature) [5,6,7,8]. This functional reinnervation was observed when nerve transfer surgeries were performed either immediately [22], after one or three months, or one year after the sacral roots of the bladder were transected [23]. This was assessed by induction of bladder contraction after electrical stimulation of the transferred somatic nerve and spinal nerve roots contributing input to the transferred somatic nerve (specifically, lumbar spinal roots in our model since we utilized somatic nerves originating from upper- to mid-level lumbar spinal cord regions). As a final confirmation of innervation, we have used retrograde dye labeling methods and observed labeled neurons in the ventral horns of lumbar spinal cord segments after injecting retrograde dye into the bladder walls of reinnervated animals [21,23].

Because of this pre-clinical success, we continue to pursue clinical translation to patients with flaccid bladders after sacral injuries or sacral chordoma removal (which injures/removes sacral motor roots and/or nerves innervating the bladder). We have previously demonstrated in several human cadaveric studies that somatic nerve transfer to vesical branches of the IHP is technically feasible using ilioinguinal, femoral, and obturator nerve branches [12,13,24]. Another group has transferred the obturator nerve to the pelvic splanchnic nerve for recovery of urination function after sacral plexus injury in patients, although the outcomes have yet to be reported [25]. Several nerve-sparing techniques and concepts for radical pelvic surgery have been proposed (such as for radical hysterectomy) [4,10,26,27,28,29]. However, further clarification of the anatomical location and relationships of the vesical branches of the IHP and pelvic ganglion (the latter for sparing purposes) is warranted to assist with their identification during laparoscopic surgical procedures for the transfer of somatic nerves to the vesical branches of the IHC for the purposes of restoration of bladder function. Thus, our primary objective was to clarify, in human cadavers, the location of vesical branches of the IHP and pelvic ganglia, their relationship to the ureteral orifice into the bladder, and their size (needed for consideration of future surgical procedures).

## 2. Materials and Methods

### 2.1. Study Material

Forty-four internal pelvic regions were dissected in 30 human cadavers (19 females and 11 males). The age range of the cadavers in which the age was known was 23 to >90 (see Table S1). Cadavers were obtained from (1) the Human Gift Registry program to Lewis Katz School of Medicine, Temple University, Department of Biomedical Education (Philadelphia, PA, USA; *n* = 21); (2) the Anatomy Gifts Registry to Nadol Surgical Skills Lab of Mass General Eye and Ear (Boston, MA; *n* = 2); (3) the Saint Louis University School of Medicine, Center for Anatomical Science and Education, Practical Anatomy Surgical Education (PASE; *n* = 3); or (4) Science Care (Phoenix, AZ, USA) to Temple University (*n* = 4). Our studies were performed in compliance with the policies of each institution and center, the National Institute of Health and the U.S. Department of Health and Human Services [30,31,32].

Nineteen cadavers had been embalmed with formalin–phenol fixative, 3 with Thiel fixative [33], and 8 were unfixed (Table S1). The 19 formalin–phenol-fixed cadavers were studied as an extension of routine anatomical dissection courses; most required additional dissection. The 3 Thiel embalmed cadavers were part of a continuing medical education course since this fixative allows for insufflation and the use of laparoscopic techniques. The 8 unfixed cadavers were specifically acquired for this study. Cadavers were inspected to ensure that the internal pelvic floor anatomy was intact. In cases where needed structures were damaged, they were not assessed (see Table 1 and Table S1). 

### 2.2. Gross Post-Mortem or Open Intra-Operative Abdominal Approach 

An anterior abdominal approach was used in 25 cadavers. The abdomen was opened. An anterior pelvic approach was used to identify the pubic symphysis, sacral vertebra, bladder, ureter, inferior hypogastric plexus (IHP), uterus (if present), and the entrance of the ureter into the bladder (i.e., the ureteral orifice). The anatomical location of the vesical nerve branches of the IHP and pelvic ganglia in relation to the ureteral orifice was assayed, as was the number of vesical nerve branches of the IHP. The width of the vesical branches, distance of the pelvic ganglia from the ureteral orifice, and the diameter of these ganglia were measured using a caliper (Instant Readout Digital Caliper, model 35.180, Grobet USA, Electron Microscopy Sciences, Hatfield, PA, USA) or a flexible ruler with millimeter markings.

### 2.3. Manual Laparoscopic or Robot-Assisted Laparoscopic Approach

Manual laparoscopic and robot-assisted laparoscopic surgery were used to gain entrance into the abdomen and pelvis of five cadavers. The laparoscope approach was as previously described [34]. The bladder, ureter, IHP, vesical branches of the IHP, and pelvic ganglia were identified.

### 2.4. Histological Assessment of Nerve Branches

Vesical branches of the IHP and pelvic ganglia were collected for histological verification (from fixed and unfixed cadavers). These nerves, ganglions, and associated soft tissues were collected as a tissue block that ranged from 2 cm to 3 cm in length and width to preserve their integrity. The block was fixed in 4% buffered paraformaldehyde (even if previously fixed) for 48 h (to rehydrate or fix), incubated in phosphate-buffered saline for 48 h, and processed and embedded in paraffin using standard methods. They were sectioned into 5-micron slices and stained with either hematoxylin and eosin or Luxol fast blue [35]. Sections were assessed microscopically using a Nikon E800 microscope and a digital camera (Jenoptik Graphax^®^ Kapella, Huntsville, AL, USA) linked to an image analysis system (Bioquant, Life Science, Nashville, TN, USA). The diameters of the vesical branches of the IHP were then quantified using the image analysis system and a 4× objective. At least three nerve sections in three non-adjacent slides were quantified for each sample.

### 2.5. Statistics

Descriptive statistics were performed using GraphPad Prism 10 or Microsoft Excel version 16.83. The mean ± standard deviation (SD), mode (the number that occurs most often in the data set), and range are provided, as appropriate for the data. All raw data are provided in Table 1 and Table 2 and Table S1.

## 3. Results

### 3.1. Vesical Nerves and Pelvic Ganglia Were Typically Located Posterolateral to the Ureteral Orifice

**Figure 1:**
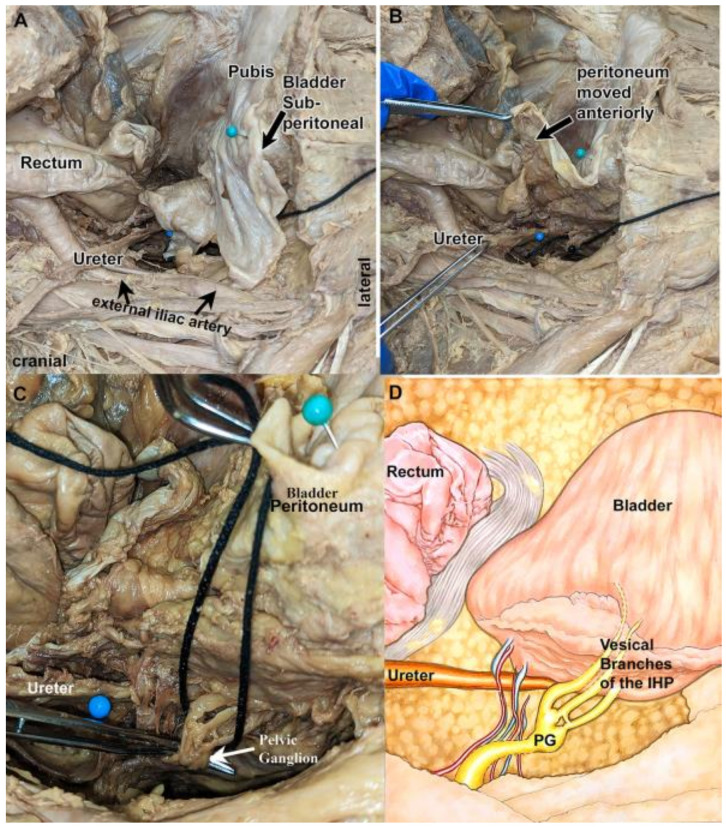
An intact pelvis showing a retropubic bladder, looking from lateral to medial. The bladder is indicated by an aqua-colored pin, ureter by a blue-colored pin, and vesical branches of the IHP are looped by black string. (**A**) Bladder is in its retropubic and sub-peritoneal location. (**B**) The peritoneum is reflected anteriorly. The ureter, immediately proximal to its entrance into the bladder, is indicated by a blue pin. (**C**) Enlarged image of ureter and pelvic ganglion (PG; white arrow). The ganglion is the slight enlargement. There are three vesical branches moving anteromedially to bladder’s surface. (**D**) A diagram of the same image as shown in panel (**C**).

First, four formalin–phenol-fixed cadavers were examined intact using an open abdominal and anterior pelvic approach (Table S1). A representative cadaver is shown in Figure 1A–C. The bladder was retropubic and sub-peritoneal in location (Figure 1A). After reflection of the peritoneum (Figure 1B) and elevating the bladder in an antero-medial direction (Figure 1C), vesical branches of the IHP and pelvic ganglion could be visualized (Figure 1C,D). The location of the vesical branches of the IHP was posterolateral to the ureter at the ureteral orifice in this cadaver (Figure 1C).

**Figure 2:**
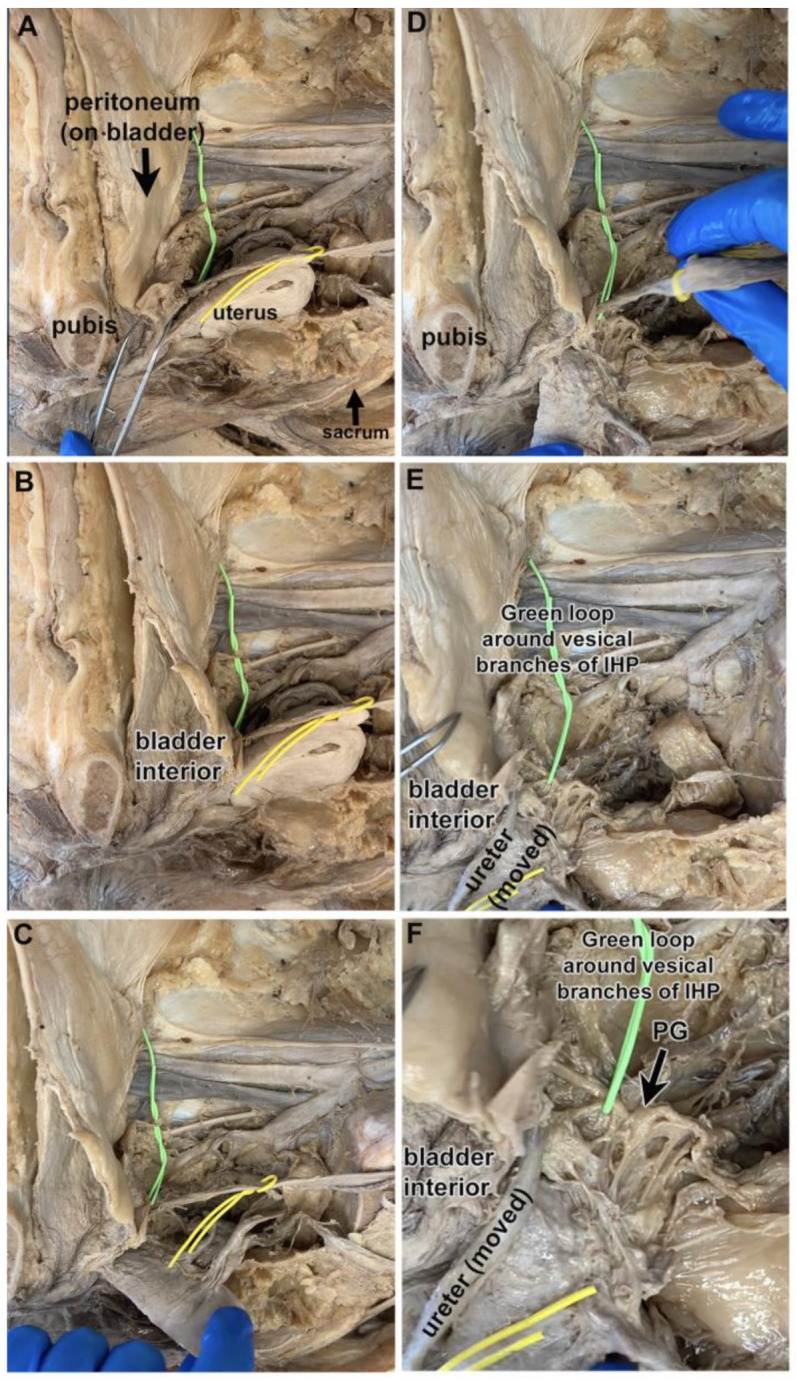
A hemisected pelvis showing a retropubic and sub-peritoneal bladder, and vesical branches of the IHP and pelvic ganglion (PG). The sacrum demarks the posterior vertebral column, while the pubis demarks the anterior medial boundary of the pelvis. The top of each image is cranial spatially in the cadaver. (**A**) Hemisected pelvis with several landmarks indicated: pubis, peritoneum-covered bladder, uterus, ureter (yellow loop), and sacrum. Vesical branches of the IHP are not yet visible (green loop). (**B**) The bladder is opened to reveal its interior. (**C**) The uterus is reflected towards the sacral vertebra. Ureter is delineated by a yellow loop; vesical branches of IHP are delineated with a green wire. (**D**) The uterus is reflected out of the field; the ureter is being reflected. (**E**) With their reflection, vesical branches of the IHP are now visible (green wire). (**F**) Enlargement of image in panel (**E**). Vesical branches are delineated by a green wire. The pelvic ganglion (PG) is labeled (large black arrow).

**Figure 3:**
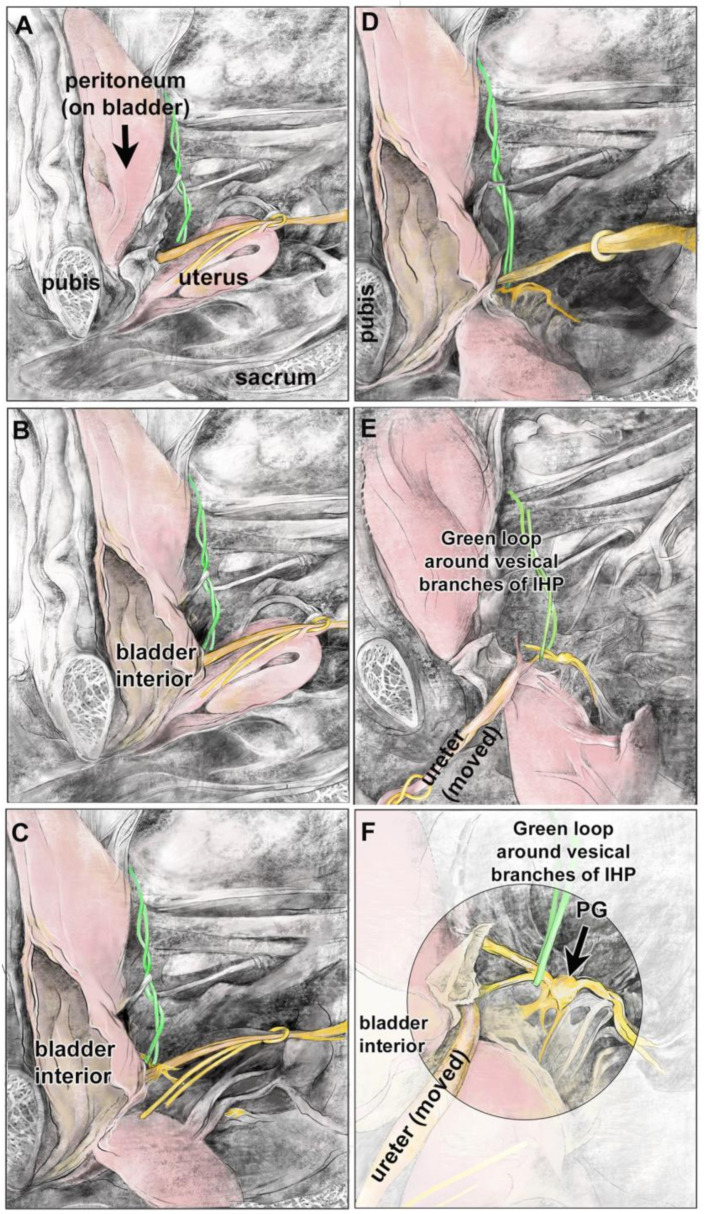
Diagrams of same panels shown in Figure 2. The sacrum demarks the posterior vertebral column, while the pubis demarks the anterior medial boundary of the pelvis. The top of each image is cranial spatially in the cadaver. (**A**–**F**) The ureter is delineated by a yellow loop; a green wire is looped around the vesical branches of the IHP, which are colored yellow for visibility in panels (**D**–**F**). Two main vesical branches are visible in panels (**E**,**F**). The pelvic ganglion (PG) is labeled (large black arrow).

Figure 2A–C shows a hemisected pelvis in which the bladder is viewed looking lateral to medial (the cut pubis, sacrum, and uterus are labeled as landmarks; the ureter is looped with a yellow vessel loop). The bladder was left attached to the pubis in its subpubic position, although opened for bladder verification (Figure 2B). The uterus and ureter were reflected to visualize the structures posterior to the ureter (Figure 2C–E). After these reflections, two vesical branches of the IHP could be identified posterolateral to the ureter’s entrance into the bladder (green loop; Figure 2E,F). These vesical branches arose from an enlarged and nearby pelvic ganglion (Figure 2F). In this cadaver, the location of the vesical branches of the IHP was lateral to the bladder and posterolateral to the ureter at the ureteral orifice. These same structures are shown as diagrams in Figure 3A–F.

The location of the vesical branches of the IHP was examined in the pelvis of 30 cadavers; 14 cadavers were examined bilaterally (Table 1). Five were fixed cadavers with intact pelvic regions, 11 were unfixed or Theil embalmed cadavers with intact pelvic regions, and 15 were fixed cadavers with hemisected pelvic regions to better access regions lateral to the bladder (Table S1). Vesical branches of the IHP could be found posterolateral to the ureteral orifice in 69% of cases (Table 1). The others were found overlying the ureteral orifice (10%), or lateral, immediately posterior, or anterolateral to the ureteral orifice (Table 1). These vesical branches typically arose from a grossly visible pelvic ganglion (93%) of cases located immediately adjacent to the bladder wall (later confirmed histologically as a neuronal ganglion). The remaining 7% of the vesical branches of the IHP were not associated with a pelvic ganglion near the bladder or ureter, but instead arose from other ganglions located nearer the sacral roots and sacrum. No sex differences were observed in the locations of vesical branches of the IHP and pelvic ganglion (Table 2).

### 3.2. Most Cadavers Had Two Vesical Nerve Branches of the IHP

**Figure 4:**
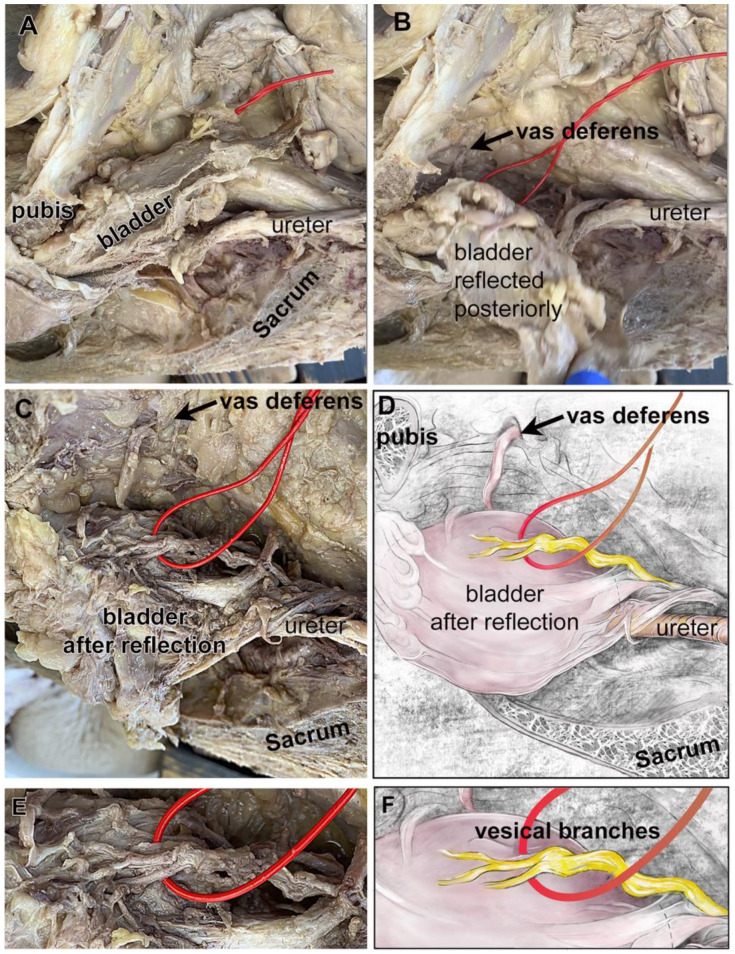
A hemisected pelvis from a male showing a retropubic bladder, and two vesical branches of the IHP. The sacrum demarks the posterior vertebral column, while the pubis demarks the anterior medial boundary of the pelvis. The top of each image is cranial spatially in the cadaver. (**A**) Hemisected pelvis with several landmarks indicated: pubis, bladder, ureter, sectioned sacral vertebra (sacrum). The vesical branches of the IHP are not yet visible (red loop). (**B**) The bladder was reflected. (**C**) With the reflection of the bladder, two vesical branches of the IHP are now visible (red wire). (**D**) Diagram of the photograph shown in panel (**D**). (**E**) Enlargement of image in panel (**C**). Two main vesical branches are delineated by a red wire, with an additional branch coming from one of the main branches. (**F**) Diagram of the photograph shown in panel (**E**).

In the cadaver shown in Figure 1, three distinct vesical branches of the IHP were observed arising from the pelvic ganglion and passing to the bladder wall (Figure 1C,D). Yet, the cadaver shown in Figure 2 and Figure 3 had two vesical branches. Examination of another cadaver (Figure 4A–F) also revealed two vesical branches arising from a pelvic ganglion (Figure 4C–F).

**Figure 5:**
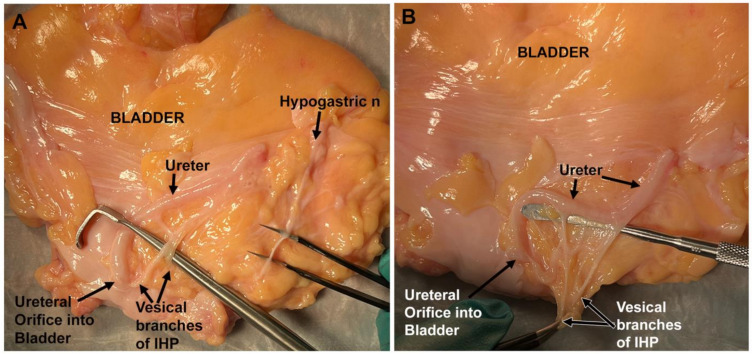
A bladder collected from an unfixed cadaver. This bladder was removed for close examination of the nerves innervating the bladder. The top of each image is cranial spatially in the cadaver. (**A**) One side of the bladder showing both vesical branches (near the ureteral orifice at the inferior-lateral base of the bladder) and a branch of the hypogastric nerve (ascending to the apex of the bladder). (**B**) The other side of this same bladder, clearly showing two main branches of the vesical branch of the IHP that then divide into multiple smaller branches, some innervating the ureter.

Figure 5 shows the vesical branches of the IHP in an unfixed cadaver’s bladder after removal of the bladder. On both the right and left sides, the vesical branches were identified posterior to the ureter on the lateral side of the base of the bladder. Two main vesical branches were identified before their subsequent further branching to the ureter and bladder wall (Figure 5A,B). Also, on each side of this bladder, prior to further branching, the main trunk of the vesical branch(es) was located near the ureteral orifice (Figure 5A,B). The hypogastric nerve was also visualized on the upper portion of the bladder, ascending to the bladder apex (Figure 5A).

**Figure 6:**
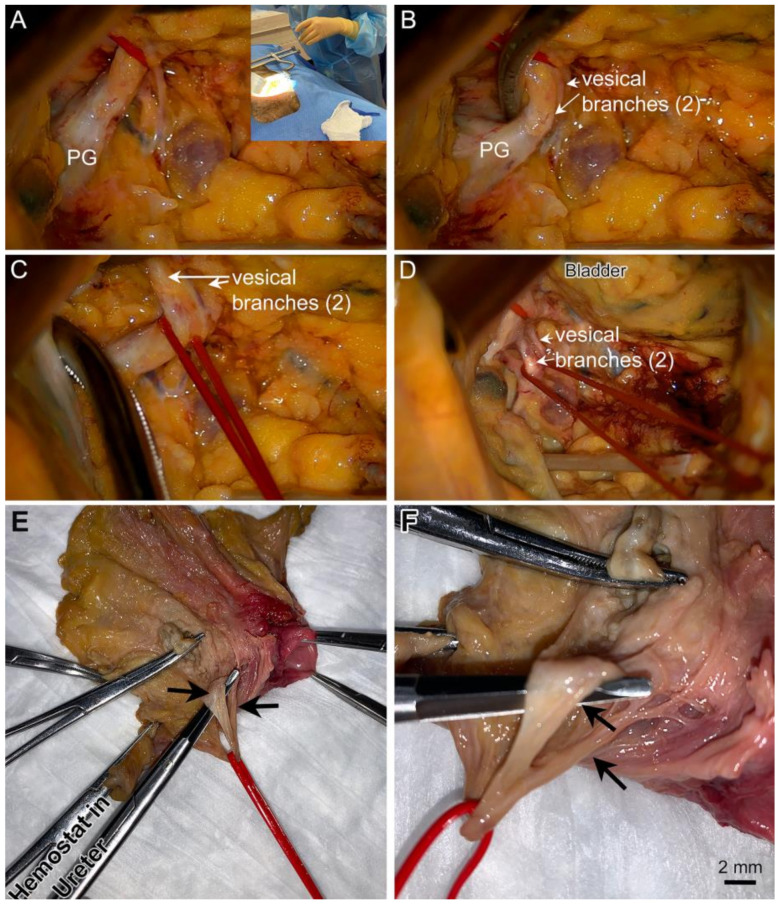
Surgical approaches were also used to examine the vesical branches of the IHP and pelvic ganglion (PG). (**A**–**D**) An abdominal approach (inset) with a surgical microscope aimed into the pelvis for visualization of pelvic structures. The image is taken looking caudally. The top of each image is anterior spatially in the cadaver. (**A**) The pelvic ganglion (PG) is visible on vesical branch of the IHP (red vessel loop), located posterolateral to the bladder within the pelvis. (**B**–**D**) Two vesical branches were exposed (white arrows). A forceps also delineates vesical branch of the IHP in panels (**C**,**D**), as does a red loop. (**E**,**F**) After laparoscopic surgery in which the vesical branches were exposed and demarked with red loops, the bladder was resected along with associated soft tissue and vesical branches. These were examined after removal and showed two vesical branches of the IHP (red loop and black arrows).

Figure 6A–D shows representative images of these structures from a cadaver captured using an intra-operative camera during a robotic-assisted laparoscopic procedure. During this procedure, the ureter was used as a key landmark. When located, the ureter was followed to the bladder. At that point, Figure 6C,D shows two vesical branches of the IHP arising from this pelvic ganglion. The vesical branches of the IHP were demarcated with red vessel loops in laparoscopic-dissected cadavers. The bladders were resected with the nerves and associated soft tissue en bloc. Figure 6E,F depict one of these resections with the red vessel loop still around the vesical branches of the IHP. Two vesical branches were identified.

Table 1 and Table 2 summarize the anatomical data regarding the number of vesical branches of the IHP. These branches ranged from 1 to 4 in number, with 2 branches being the norm (2.2 ± 0.7, mean ± SD; Table 1). When the data were divided into males versus females, no sex differences were observed in vesical branch numbers (Table 2).

### 3.3. Vesical Branches of the IHP Typically Arose Distally from a Grossly Visible Pelvic Ganglion Located near the Ureteral Orifice

Many pelvic ganglia were within the pelvic cavity, immediately posterolateral to the ureteral orifice (a location noted in 33 pelvic ganglia in 42 cadaver sides, 79%; Table 1). A few were also located overlying the ureter at the ureteral orifice, lateral, posterior, or anterolateral. The average distance of a pelvic ganglion from the ureteral orifice was 6.5 ± 6.08 mm, mostly in the posterolateral direction. The average diameter of the pelvic ganglia at its widest point was 3.8 ± 2.05 mm (Table 1), although one cadaver had a very enlarged pelvic ganglion that we suspect was a neuroma (this cadaver was excluded; neuroma shown in Figure S1). These data are similar between the sexes (Table 2), except for the distance of the left pelvic ganglion to the ureteral orifice which was less in males (3.5 ± 3.1 mm) than in females (7.9 ± 5.3 mm), although the number of males was lower than females (10 vs. 18 examined for these data), warranting caution.

### 3.4. Histological Assessment of Vesical Branches and Pelvic Ganglion

**Figure 7:**
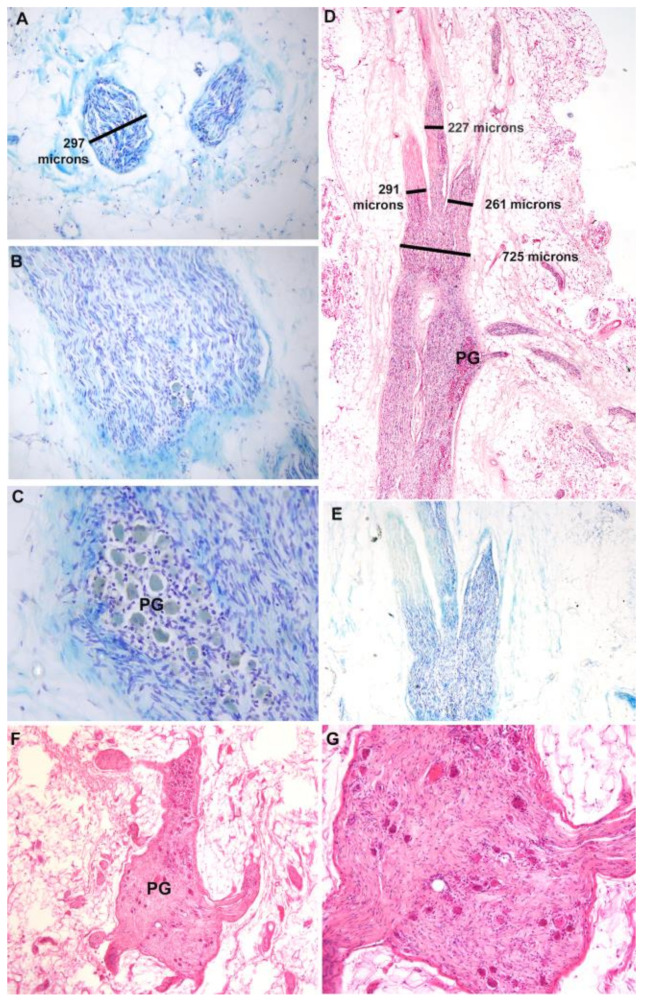
Histological images of vesical branches and pelvic ganglia (PG). (**A**–**C**) Sequential sections showing two distal vesical branches in panel A, a slightly more proximal section of the same nerve showing the presence of a few neuronal cell bodies (arrows) in panel (**B**), and a section through the pelvic ganglion (PG) of this same nerve, showing the presence of neuronal cell bodies in panel (**C**). Luxol blue staining. (**D**,**E**) Vesical branches and pelvic ganglia of a different cadaver stained with hematoxylin and eosin (**D**) and Luxol blue. Three vesical branches exit distally from the pelvic ganglion enlargement in this cadaver. (**F**) A section through a pelvic ganglion showing nerve branches and presence of neuronal cell bodies. (**G**) Enlargement of image shown in panel F. Scale markings in panels (**A**,**D**) relate also to panels (**B**,**C**,**E**,**F**).

The IHP, vesical branches of the IHP, and pelvic ganglia were collected as a block with adjacent fascial and fatty tissues from the cadavers (even the fixed cadavers), processed in paraffin, sectioned, stained with Luxol blue and/or hematoxylin and eosin, and their diameters quantified (Figure 7A–E). Two to three vesical branches of the IHP were observed arising distally from each IHP (Figure 7A,D,E), with two as the typical number as detailed in Table 1 and Table 2. The mean diameter of the individual vesical branches in these histological preparations was 0.53 ± 0.05 mm (mean ± SD) and ranged from a maximum of 1.11 mm to a minimum of 0.32 mm. The enlarged area of the nerve was confirmed as a ganglion by the presence of neuronal cell bodies (Figure 7C,F,G).

## 4. Discussion

There are currently approximately 296,000 people living with spinal cord and root injuries in the United States. Approximately 17,900 new cases of spinal injury occur annually with most resulting in tetraplegia [36]. Historically, renal failure was a commonly reported cause of death in patients with spinal injuries. With improved urogenital care, the incidence of renal failure has decreased; however, patients’ quality of life will be greatly improved with treatment for neurogenic bladder. Patients with spinal injuries frequently rank restoration of bladder function as one of the most important priorities for quality of life [2,37]. Therefore, as stated in the introduction, reinnervation strategies are crucial for either patient population and have been a topic of investigation for over one hundred years [38].

We have had success in a number of pre-clinical canine studies in restoring bladder function by transferring a somatic peripheral nerve (donor choices have included obturator, femoral, sciatic, and genitofemoral nerve branches) to vesical nerves of the IHP located adjacent to or on the bladder wall [21,22,23], which are at the distal ends of the anterior division of the inferior hypogastric plexus (IHP). We have previously demonstrated in several human cadaveric studies that somatic nerve transfer to vesical branches of the IHP is technically feasible using ilioinguinal, femoral, and obturator nerve branches [12,13,24]. However, further clarification of the location, size, and relationships of the vesical branches of the IHP and pelvic ganglion is warranted to assist with their identification during surgical procedures. Thus, this is our primary objective here.

We observed vesical branches of the IHP traveling from a nearby pelvic ganglion to the lateral wall of the bladder, mainly posterolateral to the ureter. Other branch(es) at the distal end of the IHP could be followed traveling inferiorly towards the prostate, uterus, or rectum, rather than the bladder. In this cadaveric study on 30 cadavers, typically 2 vesical branches of the IHP were found, with a range from 1 to 4 branches. The mean number of vesical branches of the IHP was similar between the right versus left sides. Some cadavers had differing numbers of branches from the left versus right pelvic ganglia, but this was a difference of only one branch. The vesical branches of the IHP have previously been described as branches at the ureterovesical junction in our previous human cadaveric studies (termed the vesical branch of the pelvic nerve in those studies) [12,13]. The course of vesical branches of the IHP has been described as consistent with two terminal branches by Mauroy et al. [5,18], matching our findings. These branches have been shown to travel with the ureter as it approaches the bladder in a recent anatomical study describing nerve-sparing radical hysterectomy methods (see Figure 16 of [10]; the vesical branches of the IHP are termed “bladder nerve branches” in that study). Another study has also reported that the vesical branches join the last segment of the ureter at the base of the bladder along its lateral border (see Figure 5 in [9]). These last two studies did not quantify the variations in the location or number of main vesical branches (nor size differences).

Although the diameter of many lower extremity nerves has been defined through many prior studies, the vesical branches of the IHP have not been equally studied in humans. We previously reported that the mean diameter of vesical branches of the IHP was 2.1 mm when measured using a caliper during anatomical dissections [24] in which the nerve branches were still covered with perineurium, fat, and other fascia. However, histological assessment of the nerves after sectioning (which allows for the distinguishment of nerve versus surrounding connective tissues and fat) revealed smaller actual nerve diameters of 0.32 to just over 1 mm (with a mean of 0.53 ± 0.05 mm). If these nerves are used as recipient nerves during surgical reinnervation of the bladder, we suggest that several smaller vesical branches of the IHP should be joined using fibrin glue, prior to surgical anastomosis to a donor nerve [39,40,41] in order to match past surgical advice that donor and recipient nerves should be similar in diameter during nerve anastomosis procedures [42,43].

Regarding the pelvic ganglia, they were typically located posterolateral to the ureteral orifice (the entrance of the ureter into the bladder), yet were also found overlying, lateral, or posterior to the ureteral orifice. The distance from the ureteral orifice to the pelvic ganglion was variable, ranging from zero millimeters (i.e., overlying) to as far as 20 mm in one cadaver, and an average distance of 7 mm. In a few cases, there were multiple ganglia on one side. Moreover, there was an asymmetry between left and right pelvic ganglion locations in a few cadavers. This is an important consideration when performing bilateral surgery, as the location and distance of the pelvic ganglion from the ureteral orifice may vary considerably between the left and right sides. There was also moderate variability in the size of the pelvic ganglia between cadavers. While the average diameter on the right versus left sides was similar, at 3.96 mm and 3.61 mm, respectively, the relatively large standard deviation of ~2 mm each is indicative of great diversity in pelvic ganglion size. The size ranged from small and diffuse ganglia to a ganglion that was 30 mm in diameter (this latter was presumed to be a neuroma; Figure S1). The size of the pelvic ganglion also varied between cadaveric sides, with a difference as large as 2.5 mm in cadaver 18. The variability in pelvic ganglion diameter may make locating the ganglion during nerve transfer more difficult in some cases. While the ureter can be used as a major landmark to reach the ureteral orifice and vesical branches [10], the medial umbilical ligaments can also be used as a landmark (personal communication from Dr. Elise De, co-author [44,45]).

It is known that injury to the pelvic autonomic nervous system innervating the bladder can contribute to long-term postoperative complications, including urinary bladder dysfunction (specifically, a flaccid bladder) and sexual dysfunction (e.g., reduced vaginal lubrication in females, reduced libido) [7,10,26,46]. Yet, very few studies have analyzed the pelvic ganglia in relation to surgery. One microanatomical study demonstrated that pelvic ganglia are prone to being damaged due to drying, hypoxia, and tension from retractors or taping during surgery [47]. Thermal damage to the ganglion from electric or ultrasonic dissection methods should also be avoided [48]. We suggest that avoiding damage to this ganglion during surgery is essential since it contains many parasympathetic post-ganglionic neuronal cell bodies responsible for bladder contraction [47].

One limitation of this study is that pelvic ganglia were stained only with hematoxylin and eosin, or Luxol blue. Future studies should also consider immunohistochemical assays using antibodies that recognize markers of the sympathetic, parasympathetic, and sensory nervous system to further describe the character of the human pelvic ganglion, as previously completed [49,50,51,52,53]. Another limitation is that we used only the ureteral orifice, ureter, and bladder as landmarks for identification and localization of the vesical nerve branches. During different radical pelvic surgeries (radical hysterectomy or prostatectomy), surgeons often use other anatomical landmarks, such as the vesicovaginal ligament, vesicovaginal venous plexus, [6,27,28,34], and medial umbilical ligaments, as mentioned earlier [44,45].

## 5. Conclusions

We have previously demonstrated the feasibility of nerve transfer procedures from somatic nerves to the vesical branches of the IHP in canines for functional reinnervation of the bladder after spinal sacral root damage, and in a total of 44 human cadavers in our past studies (which we termed vesical branches of the pelvic nerve in those studies). The 30 cadavers included in this study bring this number to 74 human cadavers in which we were able to identify vesical branches of the IHP using a variety of approaches. We extended our prior cadaveric examinations here to examine variations in the location of these distal-most branches of the IHP and their location with respect to the ureter’s entrance into the bladder wall. Clarification of the anatomy of these distal vesical branches of the IHP is critical if these nerve transfer methods are to move into clinical practice. Such clarification is also applicable to nerve-sparing concepts in radical oncological and reconstructive urogynecological surgery.

## Figures and Tables

**Table 1:** Location and number of vesical branches of interior hypogastric plexus (IHP) and pelvic ganglion (PG) per cadaver.

Cadaver Number	Vesical Branch(es) and PG Location Relative to Ureteral Orifice		# Vesical Branches of the IHP		Distance of PG to Ureteral Orifice (mm)		PG Diameter (mm)	
	Right	Left	Right	Left	Right	Left	Right	Left
1	Posterolateral	NA	2	NA	6.19	NA	3.94	NA
2	Posterolateral	Posterolateral	2	NA	10	NA	2	NA
3	Posterolateral	NA	3	NA	18	NA	8	NA
4	Posterolateral	Posterolateral	3	2	0.4	6	5	4.5
5	Posterolateral	Lateral (3.6 cm cranially on ureter)	2	4	2	5	3.50	4.20
6	NA	Lateral (overlying ureter)	NA	4	NA	5	NA	4.5 mm
7	Posterolateral	Posterolateral	3	3 (2 large, 1 small)	5	10	1 mm: several grouped ganglia	2
8	Posterolateral	Posterolateral	2 from PG; 2 non-ganglionic derived branches	1	1	13	3.5	2 and 2.3
9	Overlying (on ureteral entrance)	Overlying (on ureteral entrance)	2 (1 large, 1 very small)	3	0	0	3	3.4
10	Posterolateral	NA	2	NA	2	NA	2	NA
11	Posterolateral	Posterior (near colon)	2	2	5	15	3	3.3
12	NA (none remaining)	Main: posterolateral; 2nd: anterior to ureter	NA	1 from main ganglion	NA	Both 2	NA	3.1
13	Posterolateral	Posterolateral	2	2	2	12	30 (excluded)	2
14	NA	Overlying	NA	2	NA	0	NA	2.5
15	NA	Overlying	NA	2	NA	0	NA	2
16	NA	Anterolateral	NA	4 (1 large, 1 medium, and 2 small)	NA	7	NA	10.5
17	Posterolateral	NA	2	NA	5	NA	5.5	NA
18	NA	NA	NA	NA	NA	NA	NA	NA
19	Posterolateral	Posterolateral	4	2	11	12	3.5	3
20	Posterolateral	NA	2	NA	NA	NA	3	NA
21	Posterolateral	Posterolateral	2	2	NA	NA	8.5	6
22	Immediately posterior	Posterolateral	3	2	0.4	6 mm	NA	NA
23	Posterolateral	Posterolateral	2	2	NA	NA	3	4
24	Posterolateral	Posterolateral	3	2	NA	NA	2.5	3
25	Posterolateral	NA	2	NA	NA	NA	6	NA
26	Posterolateral	NA	2 (1 large, 1 small)	NA	NA	NA	2.1	NA
27	Posterolateral	NA	2	NA	NA	NA		NA
28	NA	NA	2	2	NA	NA	NA	NA
29	Posterolateral		1		3	2	2	1.5
30	Posterolateral	Posterolateral	2		2.5	NA	1.5	NA
Mean ± SD by side	22/23 (96%) right sides showed posterolateral location	7/19 (63%) left sides showed a posterolateral location	2.3 ± 0.6	2.4 ± 0.9	7.44 ± 7.02 mm	6.75 ± 5.17 mm	3.94 ± 2.14 mm	3.75 ± 2.28 mm
Overall ± SD	29/42 (69%) cadaveric sides showed a posterolateral location for vesical branches		2.33 ± 0.72		7.11 ± 6.11		3.8 ± 2.05	

NA = not assessed; PG = pelvic ganglia; # = number; SD = Standard Deviation.

**Table 2:** Male versus female data for vesical branches of the inferior hypogastric plexus (IHP) and pelvic ganglion (PG).

	# Vesical Branches from Right PG	# Vesical Branches from Left PG	Right PG Location Relative to Ureteral Orifice	Right PG: Distance to Ureteral Orifice	Left PG Location Relative to Ureteral Orifice	Left PG: Distance to Ureteral Orifice	Right PG Diameter	Left PG Diameter
Female *n* = 18	2.4 ± 0.6	2.1 ± 0.7	14/15 posterolateral	7.9 ± 7.3 mm	10/14 posterolateral	7.9 ± 5.3 mm	3.5 ± 1.8 mm	3.2 ± 1.3 mm
Male *n* = 11	2.1 ± 0.4	3.0 ± 1.4	6/7 posterolateral	6.2 ± 7.0 mm	2/5 posterolateral	3.5 ± 3.1 mm	4.7 ± 2.2 mm	5.4 ± 3.5 mm

# = number.

## Data Availability

All data are contained within the article and in Supplementary Material.

## Supplementary Materials

The following supporting information can be downloaded at: https://www.mdpi.com/article/10.3390/diagnostics14080794/s1, Figure S1: Neuroma engulfing pelvic ganglion and nerves on bladder wall; Table S1: Cadaver demographics.
