# Supplementary material for: Anatomical Location of the Vesical Branches of the Inferior Hypogastric Plexus in Human Cadavers
Source: Diagnostics (Basel). 2024 Apr 10;14(8):794. doi: 10.3390/diagnostics14080794 (PMC11049538; doi:10.3390/diagnostics14080794)
Supplement: Supplementary file 1 [file diagnostics-14-00794-s001.zip › diagnostics-2922255-supplementary.pdf]

Table S1. Cadaver Demographics

Cadaver #	Age	Gender	Relevant observations
1	> 90	M	Formalin-phenol fixed; Intact (not hemi-sected); Healthy appearing bladder; Ureters of normal size (bilaterally); Examined right side only; Evidence of prior pelvic surgery (e.g., surgical staples)
2	> 90	F	Formalin-phenol fixed; Intact (not hemisected); Healthy appearing bladder; Ureters of normal size (bilaterally); Focused on right side (tissue was quite hardened from the fixation)
3	78	M	Formalin-phenol fixed; Intact (not hemisected); Healthy appearing bladder; Ureters of normal size (bilaterally)
4	> 90	F	Formalin-phenol fixed; Intact (not hemisected); Healthy appearing bladder; very thin wall at ureteral orifice
5	80	F	Formalin-phenol fixed; Hemisected pelvis; Healthy appearing bladder; Ureters of normal size (bilaterally)
6	80	M	Formalin-phenol fixed; Hemisected pelvis; Healthy appearing bladder; Left ureter of normal size; No data from right hemipelvis (dissected too aggressively).
7	56	F	Formalin-phenol fixed; Hemisected pelvis; Large, healthy bladder; Ureters of normal size (bilaterally), although surrounded by an abundance of fibrotic tissue (presumed endometriosis).
8	Unknown	F	Formalin-phenol fixed; Hemisected pelvis; Healthy appearing bladder, although thick muscular wall visible; Uterus shifted to left due to golf ball-sized, encapsulated uterine fibroid
9	66	M	Formalin-phenol fixed; Hemisected pelvis; Healthy appearing bladder; Ureters of normal size (bilaterally); Slightly enlarged prostate
10	Unknown	M	Formalin-phenol fixed; Hemisected pelvis; Healthy appearing bladder; Ureters of normal size (bilaterally); No data from left hemipelvis (dissected too aggressively).
11	> 90	F	Formalin-phenol fixed; Hemisected pelvis; Healthy appearing bladder; Ureters of normal size (bilaterally); Prior hysterectomy; diverticula in distal colon
12	80	M	Formalin-phenol fixed; Hemisected pelvis; Healthy appearing bladder; Ureters of normal size (bilaterally);
13	78	F	Formalin-phenol fixed; Hemisected pelvis; Bladder slightly distended; both ureters enlarged, with R>L; Multiple small calculi in right ureter (like sand); Large neuroma in pelvic ganglion region on right side
14	89	F	Formalin-phenol fixed; Hemisected pelvis; Prior hysterectomy and ileostomy; Healthy appearing bladder; Ureters of normal size (bilaterally); unable to assess right side due to hysterectomy scar tissue
15	Unknown	F	Formalin-phenol fixed; Hemisected pelvis; Small yet healthy bladder; Ureters of normal size (bilaterally);
16	84	M	Formalin-phenol fixed; Hemisected pelvis; Very large and flaccid bladder; Unable to assess right side due to bowel compaction issues
17	86	M	Formalin-phenol fixed; Hemisected pelvis; Ureters of normal size (bilaterally); Examined right side only due to over dissection in prior anatomy class
18	85	M	Formalin-phenol fixed; Hemisected pelvis; Ureters of normal size (bilaterally); A large tumor was evident in and around bladder. Ganglion engulfed yet sacral contributions still traceable.
19	> 90	F	Formalin-phenol fixed; Hemisected pelvis; Ureters of normal size (bilaterally); Uterine fibroids; Bladder of normal size.
20	Unknown	F	Unfixed; Intact (not hemisected); Healthy appearing bladder
21	Unknown	F	Unfixed; Intact (not hemisected); Healthy appearing bladder
22	Unknown	F	Unfixed; Intact (not hemisected); Healthy appearing bladder
23	77	F	Unfixed; Intact (not hemisected); Healthy appearing bladder
24	62	F	Unfixed; Intact (not hemisected); Healthy appearing bladder
25	71	M	Unfixed; Intact (not hemisected); Healthy appearing bladder
26	Unknown	F	Thiel embalmed; Intact (not hemisected); Healthy appearing bladder
27	Unknown	F	Thiel embalmed; Intact (not hemisected); Healthy appearing bladder
28	Unknown	F	Thiel embalmed; Intact (not hemisected); Healthy appearing bladder
29	23	M	Unfixed; Intact (not hemisected); Healthy appearing bladder
30	> 90	F	Unfixed; Intact (not hemisected); Enlarged bladder

Abbreviations: F = female; M = male

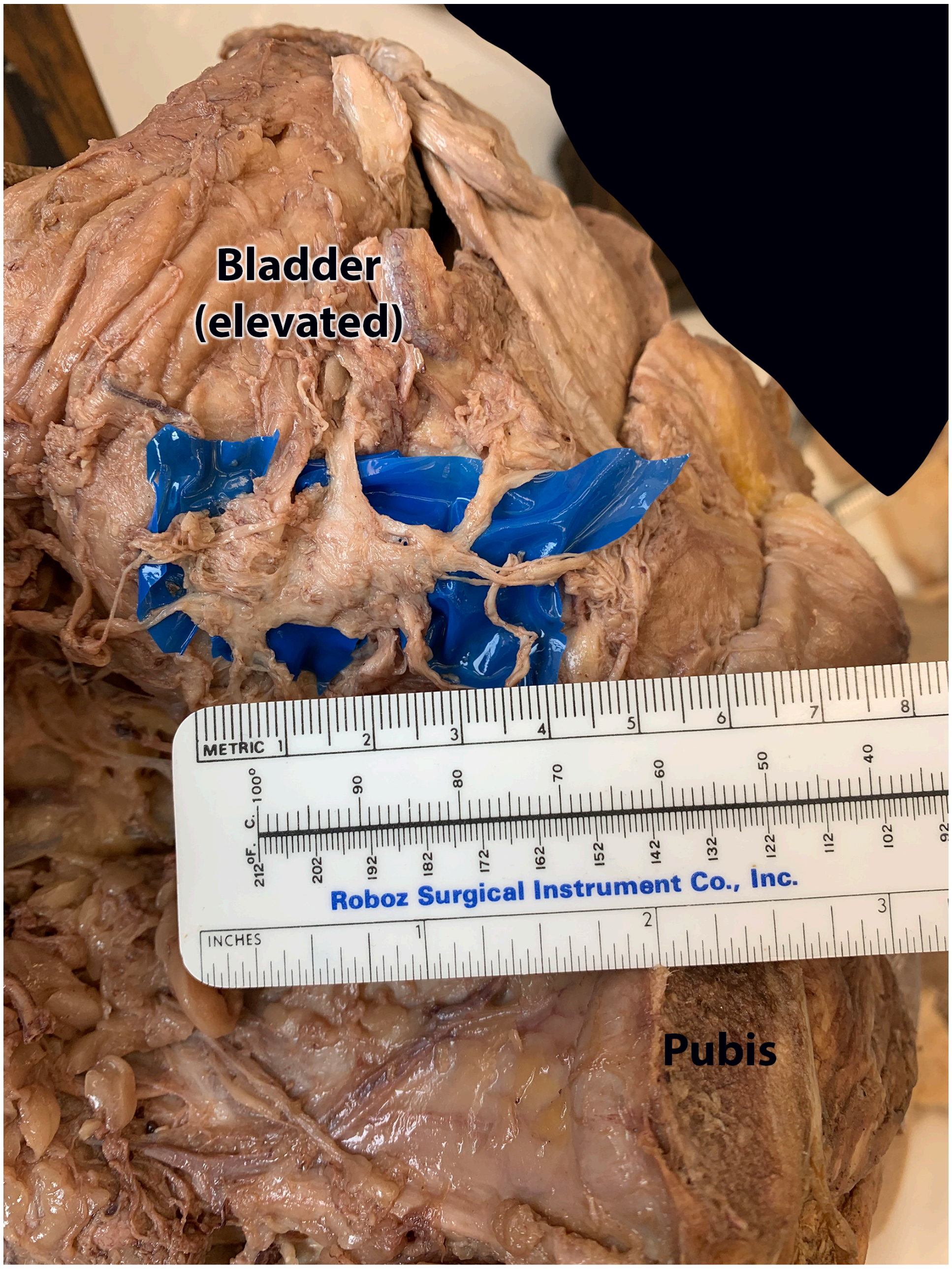

Figure S1. Neuroma engulfing pelvic ganglion and nerves on bladder wall (posterolateral to ureter).
